# Identifying a cell wall ideotype for increased soil carbon contents associated with *Miscanthus* cultivation

**DOI:** 10.3389/fpls.2025.1729614

**Published:** 2026-01-06

**Authors:** Amanda Holder, Karen Askew, Paul Robson

**Affiliations:** Institute of Biological, Environmental and Rural Sciences (IBERS), Aberystwyth University, Plas Gogerddan, Aberystwyth, Ceredigion, United Kingdom

**Keywords:** *Miscanthus*, soil carbon, composition, ideotype, bioenergy, biomass crops

## Abstract

Dedicated biomass crops are widely accepted as an important part of decarbonising economies. *Miscanthus* is a leading dedicated biomass crop that embodies high yield with many co-benefits including soil carbon sequestration plus the benefits of perennial agronomy. Breeding programmes for *Miscanthus* are in their infancy but it is apposite to consider the potential for breeding improved soil carbon sequestration. We focussed on gross biomass inputs from leaf, roots and rhizome and examined both dry weight and cell wall composition as key factors that influence soil carbon sequestration. We measured lignin, cellulose, hemicellulose, carbon and nitrogen from all three tissues in different *Miscanthus* genotypes that had been grown in field plots and compared for soil carbon. There was a significant genotypic effect on most compositional traits from all three tissue types and composition also varied between tissue types. When combined with soil C data from field grown plants, lignin and lignin:N were shown to be useful predictive factors (along with soil depth) for total soil organic C and this combination of factors explained 86% of the model variance for *Miscanthus* derived soil C. Examples of trade-offs were observed but a high sequestering plant includes low root lignin and high belowground biomass.

## 1 Introduction

Biomass is needed to decarbonise economies because it is a renewable source of complex, high energy, organic molecules that can be used for bioenergy and biorenewable materials and chemicals (Haribabu et al., 2022). Dedicated biomass crops are widely accepted as an increasingly important part of the biomass needed because production is scalable, negative emissions are possible with and without incorporation into long term products or coupled with carbon (C) capture and storage plus their cultivation often delivers many additional valuable ecosystem services (Charlton et al., 2009; Clifton-Brown et al., 2023; Smeets et al., 2009). *Miscanthus* is a leading dedicated biomass crop that embodies high yield with many ecosystem benefits plus benefits from perenniality (McCalmont et al., 2017; Robson et al., 2020). For example cultivation results in an excellent energy balance which for the standard commercial *Miscanthus* clone (*M. × giganteus*) was maximally 264–350 GJ ha^-1^ yr^-1^ over an eight-year growing period with an energy efficiency ratio of 18.6–23.3 (Dubis et al., 2019). This compares highly favourably with annual biomass crops such as maize. In a farm scale study *Miscanthus* achieved net energy production of 254 GJ ha^−1^ yr^−1^ compared with 91 GJ ha^−1^ for maize, and the related energy efficiency ratios were 5.5 (maize) and 47.3 (*Miscanthus*) (Felten et al., 2013). In this study and others *Miscanthus* was considered to be CO_2_ neutral and even a C-sink due to C sequestration to soil during cultivation and if adopted widely to replace annual crops like maize potential impacts included lower greenhouse gas (GHG) emissions, reduced nitrogen (N) leaching and higher productivity in terms of biomass yield (Davis et al., 2012).

Net sequestration of C to soil is an important and, when compared with annual crops, a largely unique co-benefit of perennial biomass crops, such as *Miscanthus* (Hansen et al., 2004; Kantola et al., 2017; Zatta et al., 2014) and along with energy balance this is an important measure of sustainability (Robson et al., 2020). Based on modelling, the average soil organic matter (SOM) accumulation rate in the top 30 cm after vegetation change from cropland to *Miscanthus* was estimated to be about 1 Mg C ha^-1^ yr^-1^ (Anderson-Teixeira et al., 2009). Soils are a vital component of global C with SOM containing more than three times as much C as the atmosphere (Batjes, 2000). It is estimated that agriculture over 1200 years has depleted soil C by as much as 133 Pg C for the top 2 m of soil (Sanderman et al., 2017). It has been argued that there is considerable potential for repletion of this lost soil C and generally greater levels of C sequestration into soils. Because most of the C in soil is autotrophically fixed by plants increasing the soil C pool is therefore a highly desirable aim for plant breeding (Kell, 2012).

It seems sensible to identify win-win scenarios, where possible, that may to some extent begin to redress the imbalance of previous crop production and the impacts of excessive industrialisation. The growth and utilisation of dedicated biomass crops can deliver such a scenario in potentially resulting in high sequestration of atmospheric C via both above- and below-ground biomass (Lemus and Lal, 2005). For example the C in above-ground biomass may be sequestered long term if the use of biomass is coupled with C capture and storage or if it is stabilised within long term products. The C in below-ground biomass may be sequestered into more stable organic forms and made inaccessible to microbes through mineral associations and/or within lower soil horizons through a range of complex interactions (Basile-Doelsch et al., 2020; Dignac et al., 2017; Poirier et al., 2018). Breeding programmes for *Miscanthus* are in their infancy but it is timely to consider both the potential for breeding *Miscanthus* for improved soil C sequestration and/or to ensure current breeding efforts do not inadvertently reduce the C sequestration potential of the crop.

C inputs to the soil environment from *Miscanthus* plants include senesced leaf, root and rhizome biomass and root exudate. But if breeding was to target improved soil C sequestration what traits would be most suited? Sequestered C accrues over many years and consequently studies of traits that impact C sequestration are relatively long term and few in number. Meta-analysis of soils within forests demonstrate the importance of plant genotype, i.e. tree type, as one significant factor impacting soil C along with soil type and temperature (Devi, 2021). There are species differences in both forest floor and mineral associated C pools (Vesterdal et al., 2013); however, the mechanisms remain largely elusive with input (Díaz-Pinés et al., 2011) as well as decomposition rates (Hansen et al., 2009; Vesterdal et al., 2008) likely to be mechanisms by which tree species influence soil C stocks.

Tree traits such as tissue chemistry and morphology impact decomposition rates, more so than climate (Hu et al., 2018) with N being positively and total C and lignin being negatively correlated with decomposition rate (Aulen et al., 2012). However, C sequestration in soils is more complex than decomposition and is a dynamic process resulting from the interaction of plant traits, climate, microbes and soil chemistry. Ultimately the aim is to achieve not decomposition of plant inputs but stable C sequestration and thus measuring soil C as an end product and relating this to trait variation is a useful approach. Plant tissue chemistry is expected to influence the stability of C within soils through different mechanisms including directly impacting the availability of C, indirectly changing physicochemical conditions within soils and altering the soil microbiome. In a 40-year common garden tree experiment differences in tissue chemistry, primarily N and recalcitrant compounds were responsible for C stability (Angst et al., 2019). The most prevalent recalcitrant compound in plants is the cell wall cross-linking polymer lignin. Lignin in soil is entirely plant derived and can form a component of the more stable mineral associated organic C pool (Whalen et al., 2022). However, depending on the favoured mechanisms, litter centred or soil centred (Poirier et al., 2018) the same litter qualities may lead to different outcomes. Perhaps reflecting the inherent complexity but also emphasising the importance of empirical determinations of soil C above inferential mechanistic studies.

Here we used a plot trial of different *Miscanthus* genotypes that had grown for ten years in a grassland-to-biomass crop conversion and determined variation in soil C (Holder et al., 2025). We grew the same genotypes in pots within a polytunnel to determine genotypic variation in total biomass across three tissue types, senesced leaf, root and rhizome. We also determined quality characteristics of the three tissue types including the contents of C, N, cellulose, hemicellulose, and lignin. We were then able to relate the quantity and quality of plant tissue inputs to longer term C sequestration within plots and identify a likely high C sequestration ideotype. With such information breeding for a high soil C sequestration type via plant tissue quality characteristics is possible because plant tissue chemistry is a highly tractable trait and has been the subject of extensive research to manipulate the energetic values of biomass (Dixon et al., 2024; Yu et al., 2021).

## 2 Materials and methods

### 2.1 Plant material and growing conditions

Rhizomes from 11 diverse *Miscanthus* genotypes (**Table 1**) were dug and split from 10-year-old field grown plants during early spring in March 2022. The rhizome was taken from a field trial based in Aberystwyth, UK, that was originally planted as part of a wider European plant trial (Kalinina et al., 2017). After splitting the rhizomes were then pared back to one bud and planted into 1 litre pots using a mix of loam-based compost (John Innes No. 3) and perlite (30%) and were grown in a polytunnel until the following spring to develop a root system. Then five replicates from each genotype were carefully washed to remove perlite and compost and potted on into 10 litre pots using sieved (1 cm mesh) compost (John Innes No. 3). Plants were randomised within the polytunnel and grown until December with manual watering according to need. Air temperature, relative humidity, and Photosynthetically Active Radiation within the polytunnel were recorded over the study period of April to December 2023 (Skye Instruments, UK) (**Supplementary Table 1**).

**Table 1:** *Miscanthus* genotypes and groupings.

Genotype ID	Species	Grouping
OPM1	*M. sacchariflorus*	*Sac*
OPM2	*M. sacchariflorus*	*Sac*
OPM3	*M. sacchariflorus*	*Sac*
OPM4	*M. sacchariflorus × M. sacchariflorus (Robustus)^1^*	*Sac×Rob*
OPM5	*M. sinensis × M. sacchariflorus*	*Sin×Sac*
OPM6	*M. sacchariflorus (Robustus)^1^ × M. sinensis*	*Rob×Sin*
OPM7	*M. sinensis × M. sacchariflorus*	*Sin×Sac*
OPM8	*M. sinensis × M. sacchariflorus*	*Sin×Sac*
OPM9	*M. sinensis × M. sacchariflorus (M. × giganteus)*	*Sin×Sac*
OPM10	*M. sinensis × M. sacchariflorus*	*Sin×Sac*
OPM11	*M. sinensis (Goliath)*	*Sin*

^1^Robustus is a M. sacchariflorus subtype.

### 2.2 Biomass harvest and sample preparation

From October until the biomass harvest which took place in December/January senesced leaves were collected from the plants before abscission. For the biomass harvest the pots were left to dry out and any remaining leaves on the plant were collected and separated into green leaves and senesced leaves. The stems were cut off at ~2.5 cm above the pot surface and removed. Below-ground biomass was gently separated from the dry compost by hand and the root and rhizome portions separated. The majority of the compost attached to the biomass was removed by hand by gently shaking and rubbing before the biomass was finally rinsed with water. The compost from each pot was then sieved (1 cm mesh) to capture any sizeable biomass remaining. Senesced leaf and separated below-ground biomass were oven dried to constant weight (40°C) to obtain the dry mass (g_dm_) and then, in preparation for laboratory analysis, representative subsamples were milled to 2 mm (Pulverisette 15 mill, Fritsch GmbH, Germany).

### 2.3 Laboratory analysis

Acid Detergent Fibre (ADF) and Neutral Detergent Fibre (NDF) were determined using the Filter Bag Technique and the ANKOM A220 Fiber Analyser (ANKOM Technology, USA) as described in ANKOM (2017a) and ANKOM (2017b). Results were expressed exclusive of residual ash. The hemicellulose content was determined by subtracting the ADF value from the NDF value. For Acid Detergent Lignin (ADL), the ADF residue was treated with 72% sulphuric acid for 3 h in a DaisyII Incubator (ANKOM Technology, USA) leaving behind the lignin fraction measured on an ash-free basis as described in ANKOM (2022). The cellulose content was determined by subtracting the ADL value from the ADF value. Analysis of the percentage carbon (C) and nitrogen (N) content was carried out on ball-milled (Labman automated preparation system) senesced leaf, rhizome and root samples using an ANCA-SL elemental analyser (Sercon Ltd, UK).

### 2.4 Statistical analysis

All means estimated with ± values reflect the standard error of the mean (SEM). Data analysis was carried out in R version 4.2.3 (R Core Team, 2023). Genotypic differences in plant traits (of biomass dry matter; C, N, lignin, cellulose, and hemicellulose contents; and ratios of C:N, lignin:N, and cellulose: hemicellulose) were tested using separate one-way ANOVAs for each biomass type (senesced leaf, rhizome, and root) and trait. The following datasets were log transformed to improve the normality of model residuals: root biomass dry matter; leaf, rhizome and root N content; rhizome cellulose content; leaf C:N ratio; and leaf cellulose:hemicellulose ratio. A square root transformation was performed on rhizome cellulose content data, and an inverse transformation on rhizome cellulose:hemicellulose ratio data. Tukey HSD *post hoc* tests (package ‘multcomp’, Hothorn et al., 2008) were used when a significant ANOVA result (p<0.05) was obtained.

To explore trait relationships with soil organic carbon (SOC, Mg ha^-1^) and *Miscanthus* derived soil carbon (C_4_-C, Mg ha^-1^) a dataset of soil C stocks from under the same, but 10-year-old field grown plants (Holder et al., 2025), used to source the rhizome for this study, was used with the plant trait results obtained in the polytunnel experiment. Full methodology for soil sampling is available in Holder et al., 2025. Akaike’s information criterion (AIC) was used for the selection of best fit linear models with fixed factors of soil sample depth increment (0-10, 10–20 and 20–30 cm), lignin content, cellulose content, hemicellulose content, C:N ratio, lignin:N ratio, and cellulose:hemicellulose ratio (R packages “nlme” (Pinheiro et al., 2017) and “MuMIn” (Barton, 2023). Separate models were used for each biomass and soil C type (SOC and C_4_-C). A square root transformation was used on C_4_-C data to improve model residuals. A Pearson’s correlation statistic was obtained for soil C and the fixed factors identified as improving model fit.

## 3 Results

### 3.1 Senesced leaf biomass and composition

#### 3.1.1 Biomass

The dry matter biomass of senesced leaf varied across the genotypes from 14.7 ± 3.0 g_dm_ (OPM11) to 85.4 ± 6.1 g_dm_ (OPM4). The senesced leaf dry matter biomass values from OPM11 biomass were significantly lower than most of the other genotypes (except for OPM1, OPM10 and OPM7); OPM4 had significantly more biomass than OPM1, OPM11, OPM10 and OPM7 (*F*_10,44_ = 7.37, *p* < 0.001, **Table 2**).

**Table 2:** Senesced leaf biomass (g_dm_) and the ratios of carbon to nitrogen (C:N), lignin to nitrogen (ADL:N), lignin to cellulose (ADL: Cell), and cellulose to hemicellulose (Cell: Hemi cell) for each *Miscanthus* genotype.

Species	ID	Biomass		C:N		ADL:N		ADL: Cell		Cell: Hemicell	
Sac	OPM1	49.42 ± 4.58	abc	77.26 ± 13.67	a	9.67 ± 1.86	a	0.16 ± 0.01	a	1.02 ± 0.02	a
	OPM2	80.40 ± 9.09	ad	158.86 ± 11.42	b	22.21 ± 2.39	b	0.17 ± 0.01	a	1.03 ± 0.02	ac
	OPM3	63.40 ± 9.15	cd	148.33 ± 6.31	b	21.78 ± 1.99	b	0.17 ± 0.01	a	1.11 ± 0.02	abc
Sin (Goliath)	OPM11	14.70 ± 2.96	b	132.20 ± 16.93	b	23.73 ± 2.99	b	0.21 ± 0.01	bc	1.19 ± 0.03	cd
Sin×Sac	OPM10	43.72 ± 5.42	bc	133.93 ± 11.66	b	20.26 ± 2.13	ab	0.18 ± 0.00	ab	1.10 ± 0.02	abc
	OPM5	53.92 ± 8.13	cd	167.12 ± 12.20	b	25.63 ± 2.87	b	0.17 ± 0.01	a	1.28 ± 0.02	bd
	OPM7	29.30 ± 6.06	bc	177.73 ± 13.61	b	22.51 ± 1.29	b	0.16 ± 0.01	a	1.09 ± 0.03	ac
	OPM8	51.50 ± 6.76	cd	129.72 ± 25.11	ab	19.38 ± 3.45	ab	0.17 ± 0.01	a	1.36 ± 0.09	d
(M×g)	OPM9	55.48 ± 3.07	cd	143.40 ± 12.48	b	21.54 ± 1.80	b	0.18 ± 0.00	ab	1.19 ± 0.06	ad
Rob×Sin	OPM6	57.16 ± 13.60	cd	112.53 ± 24.55	ab	15.89 ± 3.28	ab	0.16 ± 0.00	a	1.19 ± 0.04	cd
Sac×Rob	OPM4	85.36 ± 6.11	d	155.40 ± 9.11	b	26.13 ± 1.30	b	0.22 ± 0.00	c	1.04 ± 0.03	ac

lowercase dm means "dry matter".

#### 3.1.2 Carbon and nitrogen contents

**Figure 1:**
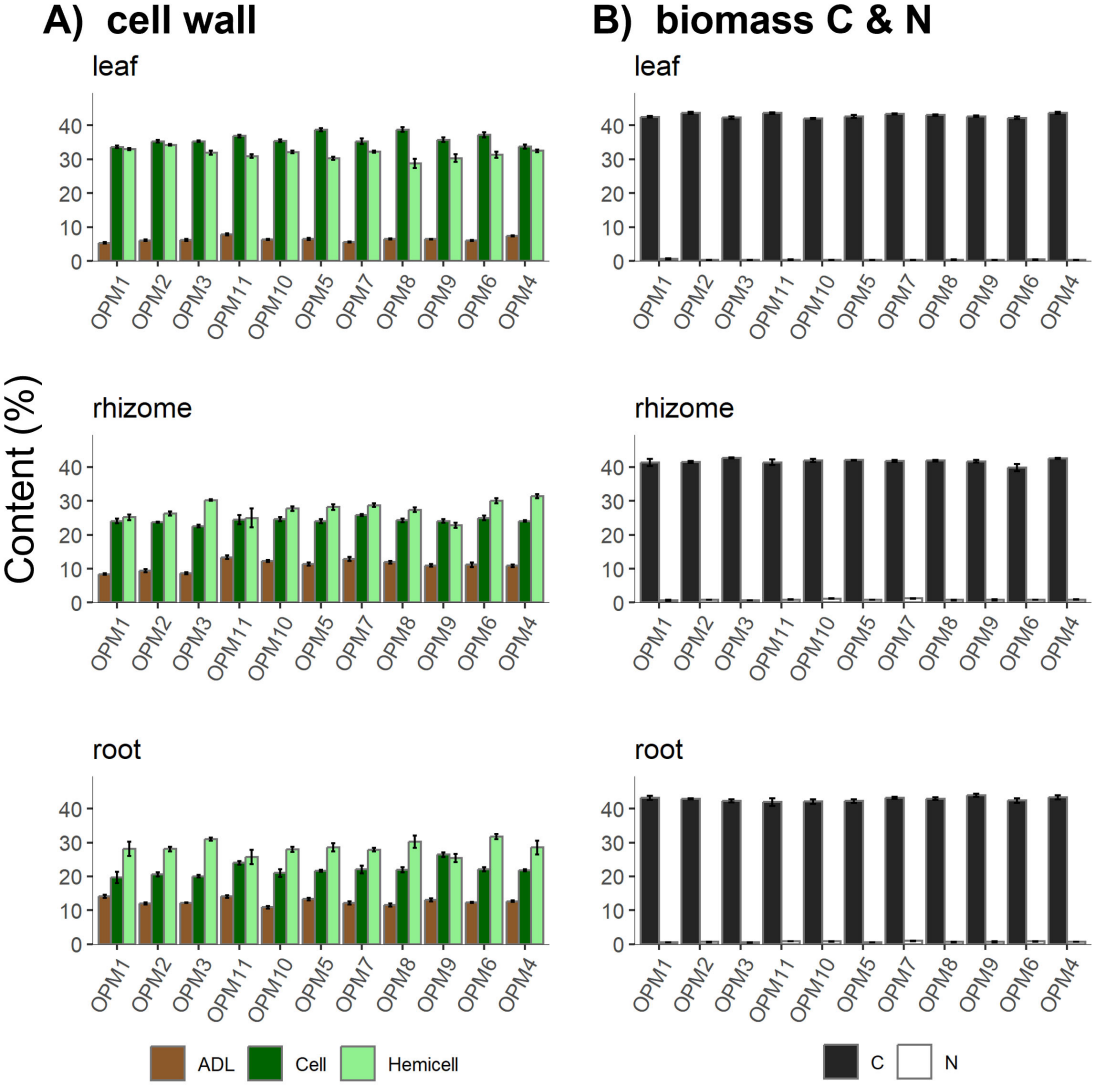
**(A)** Percentage lignin (ADL), cellulose (Cell), and hemicellulose (Hemicell) of leaf, rhizome, and root samples and **(B)** Percentage carbon (C) and nitrogen (N) of leaf, rhizome, and root samples.

The percentage C content of the senesced leaves ranged from 42% (± 0.2 from OPM10 and the same value ±0.4 from OPM6) to a significantly higher 44% (± 0.3 from OPM2, ± 0.3 from OPM4, and ±0.2 from OPM11, *F*_10,44_ = 4.88, *p* < 0.001) (**Figure 1A**). The leaf N content ranged from 0.2% (± 0.0 OPM7) to 0.6% (± 0.1 OPM1), where OPM1 was significantly higher than OPM2, OPM3, OPM10, OPM5, OPM7, OPM9 and OPM4 (*F*_10,44_ = 4.64, *p* < 0.001). The higher N content in senesced leaves from OPM1 resulted in the lowest C:N ratio (77 ± 14), which was significantly lower than the C:N ratio measured in all other genotypes except OPM6 and OPM8 (*F*_10,44_ = 4.66, *p* < 0.001, **Table 2**).

#### 3.1.3 Lignin & lignin:nitrogen ratio

The percentage lignin content in senesced leaf varied from a low of 5.3% (± 0.2 OPM1) to a high of 7.8% (± 0.3 OPM11), where OPM1 was significantly lower than OPM11 (7.8% ± 0.3), OPM5 (6.5% ± 0.3), OPM8 (6.5% ± 0.2) OPM9 (6.4% ± 0.1) and OPM4 (7.4% ± 0.1) (*F*_10,44_ = 9.84, *p* < 0.001, **Figure 1A**). The lowest lignin:N ratio was 10 (± 2 OPM1) and the highest 26 (± 1 OPM4 and ±3 OPM5), with OPM1 being significantly lower than all other genotypes except (OPM10, OPM6, and OPM8) (*F*_10,44_ = 3.72, *p* < 0.01, **Table 2**).

#### 3.1.4 Cellulose and hemicellulose contents

The percentage cellulose content of the senesced leaves was found to be between 33.6% (± 0.4 OPM1) and 38.7% (± 0.4 OPM5 and ±0.6 OPM8) (**Figure 1A**). Cellulose was significantly higher for OPM5, OPM8, OPM11 and OPM6 compared to the other genotypes (*F*_10,44_ = 10.31, *p* < 0.001). The hemicellulose content varied between 28.8% ± 1.3 (OPM8) and 34.2% ± 0.2 (OPM2), where only OPM5, OPM8, and OPM9 were significantly lower than the remaining genotypes (*F*_10,44_ = 4.71, *p* < 0.001). The cellulose to hemicellulose ratio was therefore highest for OPM8 (1.4 ± 0.1), which along with OPM11, OPM5, OPM9, and OPM6 was significantly higher than for the other genotypes (*F*_10,44_ = 7.93, *p* < 0.001, **Table 2**). The percentage cellulose content was generally higher than the hemicellulose content, except for OPM1, OPM2, OPM3, OPM10, OPM7 and OPM4 where both contents were similar (**Table 2**; **Figure 1A**).

### 3.2 Rhizome and root biomass and composition

#### 3.2.1 Biomass

Rhizome biomass ranged from 19.3 ± 4.0 g_dm_ (OPM11) to 177.4 ± 18.5 g_dm_ (OPM3). OPM11 had significantly less rhizome biomass compared to OPM1, OPM2, OPM3, OPM9, and OPM4, whereas OPM3 was significantly higher than all except OPM1, OPM9 and OPM4 (*F*_10,44_ = 28.14, *p* < 0.001, **Table 3**). Root biomass varied from 19.8 ± 3.6 g_dm_ (OPM11) to 102.3 ± 30.3 g_dm_ (OPM10). As with the senesced leaf and the rhizome biomass, OPM11 had the smallest root biomass. OPM11 root biomass was significantly smaller than all the other genotypes. The genotype with the highest weight of root biomass (OPM10) was only significantly greater than OPM11 and OPM7 (*F*_10,44_ = 11.09, *p* < 0.001, **Table 3**).

**Table 3:** Rhizome and root biomass (g_dm_) and the ratios of carbon to nitrogen (C:N), lignin to nitrogen (ADL:N), lignin to cellulose (ADL: Cell), and cellulose to hemicellulose (Cell: Hemicell) for each *Miscanthus* genotype.

		rhizome										root									
Species	ID	Biomass		C:N		ADL:N		ADL: Cell		Cell: Hemicell		Biomass		C:N		ADL:N		ADL: Cell		Cell: Hemicell	
Sac	OPM1	163.30 ± 12.26	a	67.95 ± 8.93	a	13.91 ± 1.92	ns	0.35 ± 0.02	a	0.96 ± 0.04	ab	76.02 ± 6.10	ab	75.09 ± 6.42	a	24.64 ± 2.57	a	0.74 ± 0.07	a	0.73 ± 0.10	ab
	OPM2	105.14 ± 9.80	bd	50.64 ± 2.97	ab	11.57 ± 1.05	ns	0.40 ± 0.01	ad	0.90 ± 0.02	ab	74.36 ± 2.66	ab	62.39 ± 3.14	ac	17.60 ± 1.13	ab	0.59 ± 0.01	c	0.74 ± 0.04	ab
	OPM3	177.36 ± 18.49	a	66.79 ± 7.97	a	13.42 ± 1.39	ns	0.38 ± 0.01	ae	0.74 ± 0.01	b	72.20 ± 9.82	ab	79.09 ± 5.24	a	22.90 ± 1.43	ac	0.61 ± 0.01	ac	0.65 ± 0.01	b
Sin (Goliath)	OPM11	19.30 ± 4.01	c	48.34 ± 2.47	ab	15.61 ± 0.81	ns	0.55 ± 0.03	b	1.05 ± 0.17	ab	19.75 ± 3.58	c	48.14 ± 3.24	bc	16.16 ± 0.98	bcd	0.59 ± 0.02	c	0.96 ± 0.09	ac
Sin×Sac	OPM10	67.72 ± 6.54	bc	37.10 ± 3.55	b	10.86 ± 1.09	ns	0.50 ± 0.02	bc	0.89 ± 0.04	ab	102.25 ± 30.26	a	51.75 ± 3.91	bc	13.51 ± 1.27	b	0.52 ± 0.02	bc	0.75 ± 0.04	ab
	OPM5	67.18 ± 4.04	bc	54.40 ± 2.29	ab	14.69 ± 0.94	ns	0.47 ± 0.02	bde	0.86 ± 0.05	ab	69.13 ± 4.15	ab	69.71 ± 4.04	ab	21.92 ± 0.96	ad	0.62 ± 0.02	ac	0.76 ± 0.03	ab
	OPM7	41.64 ± 3.78	c	34.38 ± 2.29	b	10.56 ± 0.87	ns	0.50 ± 0.03	bc	0.90 ± 0.02	ab	44.31 ± 8.40	b	43.74 ± 2.73	c	12.39 ± 1.04	b	0.55 ± 0.02	bc	0.80 ± 0.06	bc
	OPM8	61.00 ± 4.64	bc	56.30 ± 2.04	ab	16.04 ± 0.76	ns	0.49 ± 0.02	bd	0.89 ± 0.02	ab	78.35 ± 4.82	ab	63.95 ± 1.85	ac	17.17 ± 0.53	bcd	0.53 ± 0.02	bc	0.73 ± 0.04	ab
(M×g)	OPM9	161.62 ± 19.08	a	52.21 ± 7.31	ab	13.96 ± 2.21	ns	0.46 ± 0.02	bde	1.06 ± 0.06	a	64.91 ± 9.94	ab	63.50 ± 8.80	ac	19.10 ± 3.06	ab	0.49 ± 0.02	b	1.05 ± 0.07	c
Rob×Sin	OPM6	48.12 ± 5.77	c	48.62 ± 7.31	ab	13.53 ± 0.80	ns	0.45 ± 0.03	acd	0.83 ± 0.04	ab	62.51 ± 2.72	ab	52.59 ± 4.00	bc	15.25 ± 0.87	bd	0.56 ± 0.01	bc	0.70 ± 0.03	ab
Sac×Rob	OPM4	127.92 ± 9.80	ad	49.34 ± 4.02	ab	12.65 ± 1.24	ns	0.45 ± 0.02	cde	0.77 ± 0.02	b	51.44 ± 5.15	ab	58.75 ± 3.65	ac	17.10 ± 0.88	bcd	0.58 ± 0.01	c	0.78 ± 0.06	bc

lowercase dm means "dry matter".

#### 3.2.2 Carbon and nitrogen contents

Whilst the C content of above- and below-ground biomass was similar, the rhizome and roots generally contained more N compared to the senesced leaves. The percentage C content of the rhizomes varied (non-significantly) from 40% ± 1.1 (OPM6) to 43% (± 0.2 OPM3 and ±0.1 OPM4) (**Figure 1B**). Rhizome N content ranged from a low of 0.7% (± 0.1 OPM1, ± 0.1 OPM3, ± 0.0 OPM8) to the significantly higher 1.2% ± 0.1 (OPM10 and OPM7, *F*_10,44_ = 5.12, *p* < 0.001). Due to the higher N content in OPM10 and OPM7 rhizomes their C:N ratios were significantly lower than the two highest (OPM1 and OPM3) (*F*_10,44_ = 4.46, *p* < 0.001, **Table 3**). As with the rhizomes, the C content of the roots did not vary significantly and ranged between 42% (± 0.4 OPM3, ± 1.1 OPM11, ± 0.7 OPM10, ± 0.5 OPM5, and ±0.7 OPM6) and 44% (± 0.5 OPM9) (**Figure 1B**). Root N content varied from a low of 0.7% (± 0.1 OPM1, ± 0.1 OPM3, ± 0.0 OPM8) to a significantly higher 1.2% (± 0.1 OPM10, ± 0.1 OPM7) (*F*_10,44_ = 6.47, *p* < 0.001). The root C:N ratio was therefore significantly lower for OPM10 and OPM7 compared to OPM1 and OPM3 (*F*_10,44_ = 5.74, *p* < 0.001, **Table 3**).

#### 3.2.3 Lignin & lignin:nitrogen ratio

The lignin content of rhizome and root samples was generally higher than that found in the senesced leaves (**Figure 1A**). The percentage lignin content of rhizome ranged from 8.4% ± 0.3 (OPM1) to 13.4% ± 0.6 (OPM11) (**Figure 1A**). The lignin content was significantly less for OPM1 compared to the other genotypes (except for OPM2 and OPM3), and OPM11 was significantly higher than OPM1, OPM2, OPM3, OPM9, OPM6 and OPM4 (*F*_10,44_ = 13.25, *p* < 0.001). However, the lignin:N ratio did not vary significantly among the genotypes but ranged between 11 ± 1 (OPM10 and OPM7) and 16 ± 1 (OPM11 and OPM8) (**Table 3**). The rhizome lignin content was generally higher than the root lignin content, which was particularly noticeable for the three Sac species (OPM1, OPM2, OPM3) (**Figure 1A**). Root lignin content varied from a low of 10.9% ± 0.4 (OPM10) to a high of 14.1% ± 0.4 (OPM1 and OPM11) (**Figure 1A**). The high lignin content in the roots of OPM11 mirrored the high lignin content in the rhizomes but this relationship was not present in all genotypes. OPM1, for example, had a low lignin content in the rhizome (compared to the other genotypes) but had one of the highest lignin contents in the roots. The root lignin content from OPM10 was significantly lower than OPM1, OPM11, OPM5, and OPM9, whereas the content from OPM1 and OPM11 was significantly higher than OPM2, OPM3, OPM10, OPM7 and OPM8 (*F*_10,44_ = 7.22, *p* < 0.001). The root lignin:N ratio, varied significantly from 12 ± 1 (OPM7) to 25 ± 3 (OPM1). OPM7 and OPM10 had a lower root lignin:N ratio compared to OPM1, OPM3 and OPM5 whereas OPM1 had a higher ratio compared to OPM10, OPM11, OPM4, OPM6, OPM7, OPM8 (*F*_10,44_ = 6.37, *p* < 0.001, **Table 3**).

#### 3.2.4 Cellulose & hemicellulose contents

The cellulose and hemicellulose content of the senesced leaves was typically higher than the content of the rhizome and roots (**Figure 1A**). The cellulose content of the rhizome ranged (non-significantly) from 22.5% ± 0.4 (OPM3) to 25.8% ± 0.4 (OPM7). There was, however, more genotypic variation in the hemicellulose content of the rhizome which was found to be between 22.8% ± 0.8 (OPM9) and 31.4% ± 0.6 (OPM4) (**Figure 1A**). The hemicellulose content of the rhizome from OPM9 was significantly lower than OPM3, OPM7, OPM6, and OPM4 whereas that from rhizome of OPM4 was significantly higher than OPM1, OPM2, OPM11, OPM8, and OPM9 (*F*_10,44_ = 6.19, *p* < 0.001). Contrary to the senesced leaf cellulose to hemicellulose content, rhizome hemicellulose content was generally higher than the cellulose with the exception of OPM1, OPM11, and OPM9 where the ratio was near 1 (**Figure 1A**; **Table 3**). The lowest cellulose to hemicellulose ratio was 0.7 ± 0.0 (OPM3) which was significantly lower than the 1.1 ± 0.1 of OPM9 (*F*_10,44_ = 2.70, *p* < 0.05, **Table 2**). Root cellulose content varied from 19.7% ± 1.7 (OPM1) to 26.4% ± 0.7 (OPM9). The root cellulose contents of OPM9 and OPM11 were significantly higher than OPM1 (*F*_10,44_ = 5.31, *p* < 0.001, **Figure 1A**). The root hemicellulose content did not vary significantly, ranging from 25.4% ± 1.2 (OPM9) to 31.7% ± 0.7 (OPM6). In a similar way to the hemicellulose content from the rhizomes, the root hemicellulose content was generally higher than the cellulose, again with the exception of OPM11 and OPM9 with ratios of 1. OPM3 again had the lowest cellulose to hemicellulose ratio (0.6 ± 0.0) which was significantly lower than OPM11 and OPM9 (*F*_10,44_ = 4.00, *p* < 0.001, **Table 3**).

### 3.3 Soil C and trait relationships

When combined with soil C data from the same genotypes grown in the field, lignin content and the lignin:N ratio were shown to be useful predictive factors (along with soil depth) in best fit model selection suggesting the importance of these traits for soil C. The other traits considered (C:N ratio, cellulose, hemicellulose, and cellulose:hemicellulose ratio) did not show any relationship with the soil C data.

**Figure 2:**
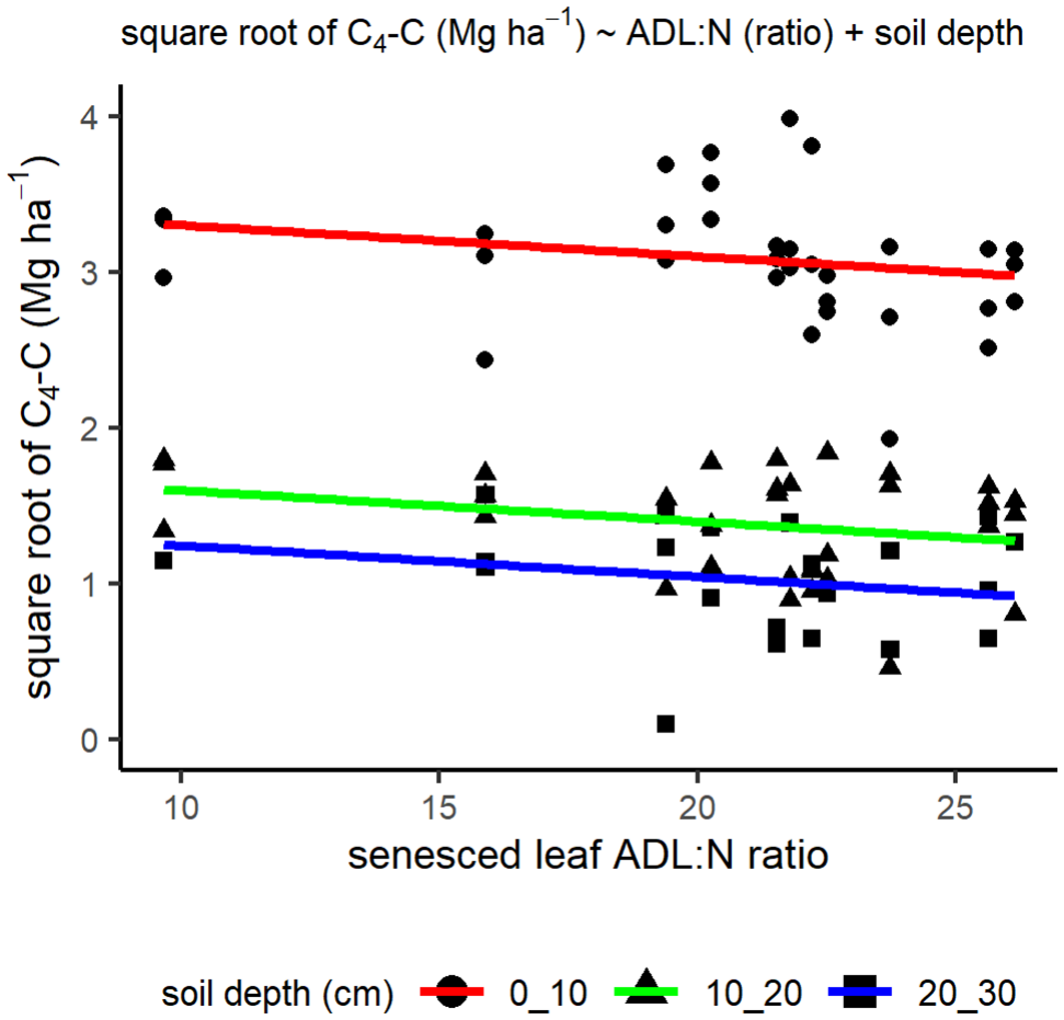
Senesced leaf lignin:N ratio from pot grown plant samples and soil C_4_-C (Mg ha^-1^) stocks from the same genotypes grown in the field at 10 years old. The C_4_-C data underwent a square root transformation to improve the normality of the model residuals. The trend lines show the model prediction.

Senesced leaf lignin:N ratio, along with soil depth were selected as important factors for predicting soil C_4_-C, explaining 86% of the model variance (**Figure 2**). However, no significant correlation was found between the lignin:N ratio and C_4_-C (r_31_ = -0.22, p = 0.22; r_31_ = -0.29, p = 0.11; r_19_ = -0.20, p = 0.39, for soil depths 0–10 cm, 10–20 cm and 20–30 cm, respectively). None of the leaf traits included were found to be important in predicting SOC.

**Figure 3:**
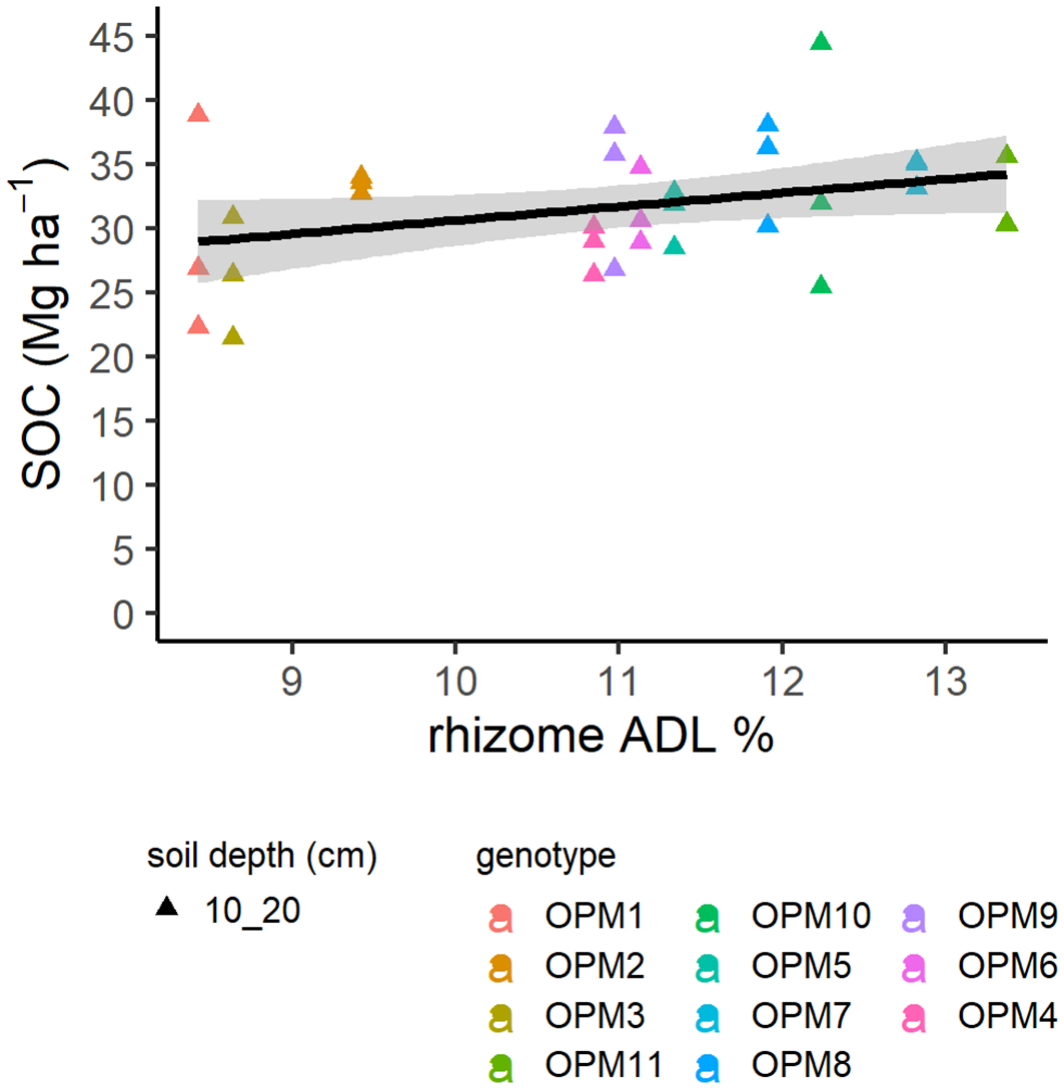
Relationship of rhizome lignin percentage content (ADL) from pot grown plant samples and soil organic carbon (SOC) at the 10–20 cm soil depth from the same genotypes grown in the field at 10 years old. The shading reflects the standard error.

Rhizome lignin content and soil depth were key factors in relation to SOC, although only predicting 55% of the model variance. However, in addition to this, rhizome lignin content was positively and significantly correlated to SOC at the 10–20 cm soil depth (**Figure 3**) (r_31_ = 0.02, p = 0.91; r_31_ = 0.34, p = 0.05; r_19_ = 0.14, p = 0.54, for soil depths 0–10 cm, 10–20 cm and 20–30 cm, respectively). No association was found between the rhizome traits considered and soil C_4_-C.

**Figure 4:**
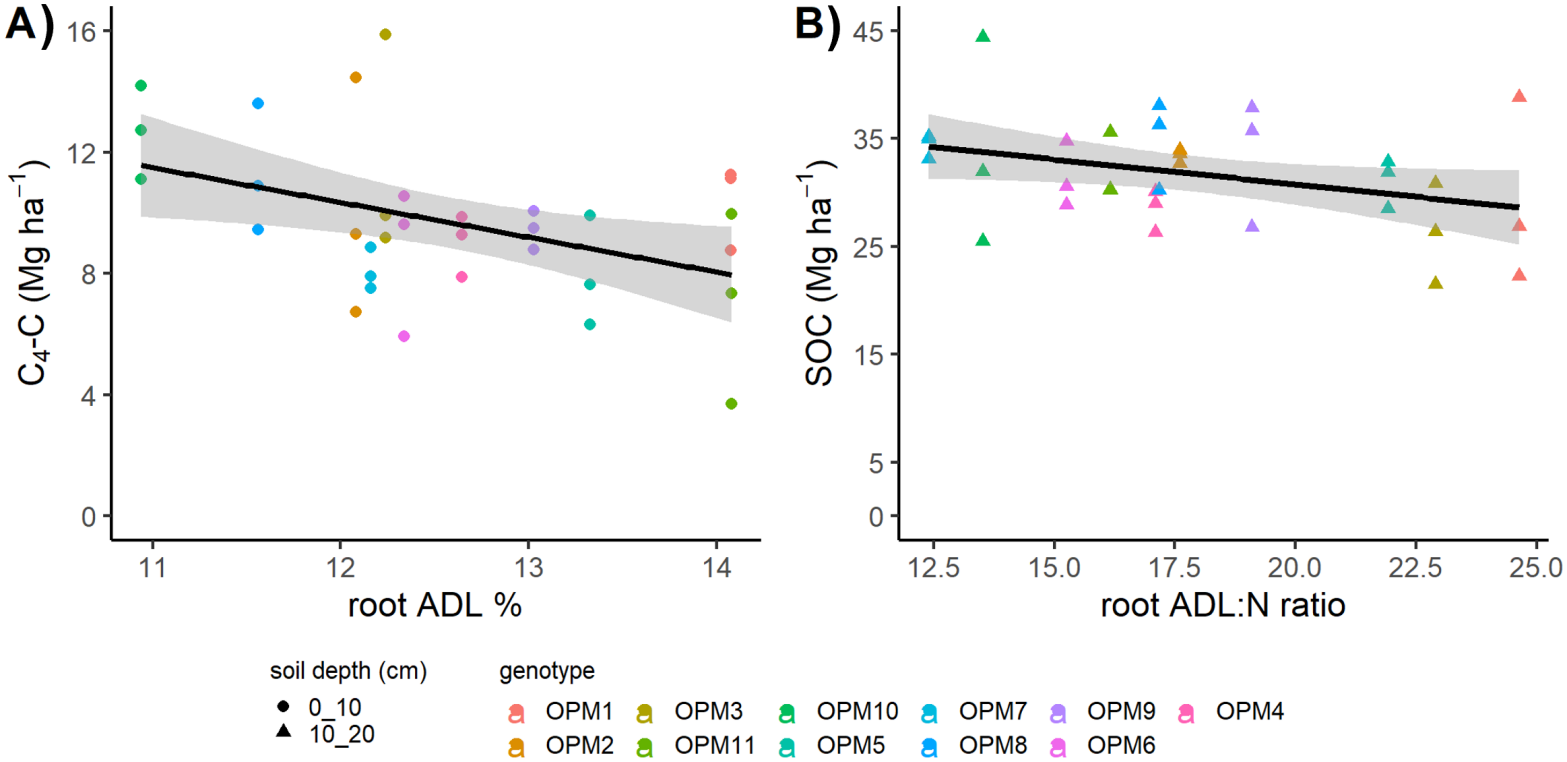
The relationship between *Miscanthus* traits from pot grown plant samples and soil C stocks from the same genotypes grown in the field at 10 years old: **(A)** root percentage lignin (ADL) content and soil C_4_-C stocks (Mg ha^-1^) at the 0–10 cm soil depth; **(B)** root lignin to nitrogen ratio (ADL:N) and soil organic carbon (SOC) stocks (Mg ha^-1^) at the 10–20 cm soil depth. The shading reflects the standard error.

Root lignin content and the lignin:N ratio, along with soil depth, were also identified as important factors in predicting soil C_4_-C, with this combination of factors explaining 86% of the model variance. Root lignin content was negatively and significantly correlated with soil C_4_-C for the 0–10 cm soil depth (**Figure 4A**), (r_31_ = -0.42, p = 0.01; r_31_ = 0.18, p = 0.31; r_19_ = -0.10, p = 0.66, for soil depths 0–10 cm, 10–20 cm and 20–30 cm, respectively), but no significant correlations were found for soil C_4_-C and the lignin:N ratio. However, a relationship was found between root lignin:N ratio and SOC, which along with soil depth was selected as a key model predictor explaining 56% of the model variance. The root lignin:N ratio was also negatively and significantly correlated with SOC at the 10–20 cm soil depth (**Figure 4B**) (r_31_ = 0.01, p = 0.96; r_31_ = -0.35, p = 0.04; r_19_ = -0.13, p = 0.58, for soil depths 0–10 cm, 10–20 cm and 20–30 cm, respectively).

## 4 Discussion

### 4.1 Trait differences and implications for breeding

The results of this study provide much needed data of *Miscanthus* cell wall composition of senesced leaves, rhizome, and roots. Currently very little is known about below-ground biomass traits for *Miscanthus* genotypes, how they differ, their relevance for *Miscanthus* breeding, or how they could potentially impact soil C cycling.

All the *Miscanthus* genotypes we examined had below-ground biomass considered to be recalcitrant compared to annual biomass crops, but less so compared to woody crops, and more similar compared to other perennial herbaceous crops. For example, the root lignin content we recorded for the *Miscanthus* genotypes (11%-14%) is generally lower for maize (*Zea Mays*, 4%-10%) (Machinet et al., 2011), higher for poplar (*Populus* spp. ~18%) and similar for Switchgrass (*Panicum virgatum*, 13%) (Ferrarini et al., 2022).

For all the *Miscanthus* genotypes included in this study above- (senesced leaves) and below-ground (rhizome and root) biomass differed in terms of N, lignin, cellulose and hemicellulose content. The lower N content of the senesced leaves reflected the nutrient translocation from leaves into plant storage organs (Magenau et al., 2022). Significant *Miscanthus* genotypic variation in cell wall composition was also found in each of the tissue types.

Significant genotypic variation was found for all the senesced leaf traits explored providing scope for breeding. Differences in *Miscanthus* leaf trait (including nutrient content) relationships between triploid and tetraploid genotypes have been identified (Li et al., 2022) showing the potential for polyploidization for desired leaf traits. In other species cell wall traits are amenable to breeding and many studies have included GWAS to understand the genetic architectures of lignin, cellulose and hemicellulose for breeding programmes (Esposito et al., 2022; Panahabadi et al., 2022; Yang et al., 2019).

The rhizome mass recorded for M×g and Sac×Rob were similar to the higher rhizome mass recorded for the three Sac species. In the Sac species root lignin content was also generally higher than rhizome lignin whereas for the Sin species and hybrids it was similar. But with the exception of this none of the hybrids showed any other traits that could be particularly linked to the Sac or the Sin species.

In the case of studies of soil organic carbon accumulation with *Miscanthus* crops they traditionally occur over many years with samples taken from mature field crops between 3 to 20+ years old (Qin et al., 2016; Zang et al., 2018). Whereas, mostly, morphology and composition traits have been assessed across shorter time scales and often in pots. It is therefore worth considering if there is a reasonable expectation that the traits measured in the pot experiment will be consistent, at least in terms of rank order, with the same traits expressed over many years in the field. For some cell wall traits such as lignin many experiments have manipulated composition and genotypes engineered for high lignin phenotypes in the pot, for example, are also high lignin genotypes in the field (De Meester et al., 2022). The environment and plant age can impact lignin content (da Costa et al., 2014), for example high temperatures (Crivellaro and Büntgen, 2020) and other abiotic and biotic stresses (Cesarino, 2019) affect lignin accumulation. Small variations have been seen in contents for above-ground biomass for these genotypes when grown in different European locations (Kiesel et al., 2017) but generally the control of lignin content is moderate to highly heritable (Harman-Ware et al., 2021; Mandrou et al., 2012; Panahabadi et al., 2022). This is consistent with cellulose, hemicellulose and lignin contents analysed from a diversity panel of *Miscanthus*, where all composition measurements had a similar and high broad sense heritability with an average across all cell wall traits of 0.67 (Slavov et al., 2013).

Contrary to results obtained for the above-ground harvested biomass (mainly stems) (Allison et al., 2011; Hodgson et al., 2010; Kiesel et al., 2017), we did not find the senesced leaf for the common commercial clone M×g (OPM9) to be higher in cellulose or lignin compared to the Sac and Sin species. However, M×g was among the lowest in terms of senesced leaf hemicellulose content as was the case for the harvested material from other studies (Allison et al., 2011; Kiesel et al., 2017). The lignin content of senesced leaf biomass (grown in Germany and separated from field harvested material in spring) for the Rob×Sin (OPM6) and Sin (OPM11) genotypes was recorded as higher than that for the Sac species (OPM3) and M×g (Schäfer et al., 2019). similar to this, in this study we also found the Sin senesced leaf to be one of the highest in lignin content, but this was not significantly higher than either the Sac (OPM3) or M×g. Both Hodgson et al. (2010) and Schäfer et al. (2019) found harvested biomass (stems and separated leaves) from the Sin species to have higher portions of hemicellulose to cellulose compared to the Sac species and M×g. In this study we also found the content comparable to two out of the three Sacs (OPM1, OPM2) and Sac×Rob (OPM4). However, in our results M×g was similar to Sin in its proportion of hemicellulose to cellulose.

### 4.2 Relationships with and implications for soil carbon

Some trait differences between above- and below-ground biomass are likely to have contrasting effects for SOC accumulation. In particular, the Sac species OPM1 had traits that could decrease decomposition i.e. high root lignin content and lignin:N ratio (compared to the other genotypes) (Abiven et al., 2005), but conversely senesced leaf for this same genotype had a 1:1 cellulose to hemicellulose ratio, the lowest lignin content, and the lowest lignin:N ratio. OPM1 leaf was also among the lowest in terms of C:N ratio, the latter being all traits that could increase decomposition (Poirier et al., 2018).

Counteracting traits were also noted within the below-ground biomass samples. The Sac species OPM3 rhizome and roots, for instance, had one of the highest C:N ratios compared to the other genotypes, a trait generally linked to slower decomposition (Nicolardot et al., 2001). But OPM3 was also among the lowest in terms of cellulose:hemicellulose ratio, a trait expected to increase decomposition (Kögel-Knabner, 2002). Diverging traits were also found between rhizome and root samples. For example, OPM1 had the lowest rhizome lignin content, but was also one of the highest in terms of root lignin content.

It could be expected that the Sin species (OPM11), with the least biomass amount, one of the highest biomass lignin contents, and a below-ground biomass cellulose to hemicellulose ratio of 1, would result in lower SOC stocks compared to the other genotypes. However, results from SOC sampling of the same 10-year-old field grown genotypes used to provide the rhizome material for this study did not show this to be the case. Following soil C sampling pre-planting and after 10 years of *Miscanthus* growth, long-term SOC stocks under the Sin plots were found to be similar to the other genotypes (Holder et al., 2025).

Of the traits considered here (lignin, cellulose, hemicellulose content, and C:N ratio, lignin:N ratio, and cellulose:hemicellulose ratio) only lignin and the lignin:N ratio were identified as important in predicting SOC and C_4_-C. Previously high lignin concentrations in plant litter have been considered to increase SOC stocks due to the slower decomposition of complex lignin compounds by only a few organisms (Bollag et al., 1997; Hall et al., 2020). And in this study, we found higher rhizome lignin content to be associated with higher SOC stocks (for the 10–20 cm soil depth). However, recent work on lignin decomposition has shown that high lignin concentrations in plant litter can have contrasting affects for SOC stocks and does not always lead to increased SOC (Hall et al., 2020; Poirier et al., 2018). In support of this and in contrast to the relationship between rhizome lignin concentration and SOC, we identified low root lignin concentration (for the 0–10 cm soil depth) and low root lignin:N ratios (for the 10–20 cm soil depth) to be associated with higher SOC and C_4_-C stocks. Low senesced leaf lignin concentration was also linked with higher C_4_-C stocks. This type of result has also been found for other C_4_ grass species, where in a comparison of tropical perennial grasses root lignin concentration was found to be a driver of SOC, with lower root lignin content varieties accumulating the greatest SOC (Sumiyoshi et al., 2017). The lower lignin litter decomposed faster, but the residue and associated microbial by-products increased SOC (Sumiyoshi et al., 2017).

Previous studies have found M×g rhizome to have a faster decomposition rate compared to roots which has been attributed to the rhizomes higher sugar content (cellulose and hemicellulose) and lower lignin content (Amougou et al., 2011; Beuch et al., 2000; Ferrarini et al., 2022; Ridgeway et al., 2022). This is likely to contribute to the different relationships of rhizome and root with SOC stocks that we found. The genotypes we explored all had higher cellulose contents in the rhizome compared to their roots with the exception of M×g (where it was slightly less) and the Sin species (where it was the same). Soil acidity, soil N availability, and climate also have a variable influence on bacteria and fungi involved in lignin decomposition (Thevenot et al., 2010). The low pH (5.3) of the soil in this study was optimal for fungal activity, but low for bacterial degradation of lignin (Thevenot et al., 2010).

The C:N ratio of *Miscanthus* root litter has also been shown to influence SOC cycling where higher substrate C:N ratios reduce microbial carbon use efficiency (CUE) and the low N limits microbial growth, requiring microbes to mine for N, thereby increasing their respiration (Ferrarini et al., 2022; Poeplau et al., 2023; Ridgeway et al., 2022). In an incubation study using *Miscanthus* root litter it was found that litter with a C:N ratio of 85 negatively affected change in SOC more than litter with a C:N ratio of 50 (Poeplau et al., 2023). The root C:N ratio of the genotypes in this study ranged from 44 to 79, a difference large enough to potentially influence C cycling. It should also be noted that C:N ratios of root, and rhizome in particular, vary seasonally due to nutrient translocation (Heaton et al., 2009; Poeplau et al., 2019).

Although lignin content is identified as an important component of SOM formation (Stewart et al., 2015) other studies emphasise the importance of monomeric composition of lignin, in particular the ratio of Syringyl to Guaiacyl subunits in lignin degradation (Aswin et al., 2025). The impacts of lignin recalcitrance and association with soil mineral particles and the susceptibility of lignin to degradation by microbes leading to the production of microbial necromass are both routes to the stabilisation of plant-derived C in soil. The relative importance of plant biomass and microbial necromass to the production of SOM remains a subject of debate (Whalen et al., 2022) and the relative importance and interactions of the substituent components of this complex nexus of soil, plant and microbiome (Basile-Doelsch et al., 2020) remain to be elucidated.

Plant litter cell wall composition, although a principal component of soil C cycling, is subject to a number of interacting factors that can modify its influence on SOC stocks. Therefore, the relationships with SOC and the genotypic trait differences observed here may have a more pronounced or different impact on SOC accumulation in different locations with varying soil conditions (e.g. pH, soil C:N ratio), climate (e.g. temperature, precipitation), and management (e.g. fertilisation). Whilst this paper provides much needed data on the composition and genotypic variation of *Miscanthus* tissue entering soil C cycling systems, the quantity of C input from the turnover of rhizome and root in the field is largely unknown for both M×g and novel hybrids and remains an important metric needed to consider the contribution of *Miscanthus* genotypes to SOC accumulation. Research directed at a better understanding of the interaction of the traits under different field conditions, and the relevant impacts on various longer and shorter-term soil C pools would also aid in identifying trait preferences for the cell wall ideotype targeted to increased soil carbon.

Our research demonstrates useful variation in the composition of above- and below-ground tissues within a small population of Miscanthus genotypes. Some of the variation is likely to be counteracting for example the genotype OPM3 had within the same tissues a C:N ratio likely to reduce decomposition and a cellulose:hemicellulose ratio expected to increase decomposition. This may explain in part the general similarity between SOC stocks in the field trial and suggests it may be more informative to identify genotypes that accumulate SOC quicker i.e. over a shorter time span. The sometimes inconsistent effects of plant inputs when many other soil related factors are similar may be explained by complex interactions such as the level of soil carbon saturation, the unsatisfied ability of different soils to store carbon (Castellano et al., 2015). The field used in the SOC study is representative of the largest category of agricultural land classification in the UK (Class 3) (Welsh Govt., 2019), and therefore is quite broadly applicable, but it would be useful to examine if variation in SOC within different soil types and or classifications give similar results. Previously we showed that high yielding *Miscanthus* genotypes did not incur a soil carbon penalty (Holder et al., 2025), which is good news for breeding programmes that focus on above-ground harvestable yield. If in addition to harvestable yield breeding programmes wish to maximise carbon sequestered into soil our results suggest a high SOC accumulating ideotype includes low root lignin and lignin:N ratio and high below-ground biomass, all of which represent significant components in our models relating traits to SOC.

## Data Availability

The data that support the findings of this study are openly available in “Pure”, doi: 462 10.20391/66592987-4d9d-4bbb-a936-928c35d0ae95 “Miscanthus biomass cell wall composition”.
