# Supplementary material for: Identifying a cell wall ideotype for increased soil carbon contents associated with Miscanthus cultivation
Source: Front Plant Sci. 2026 Jan 6;16:1729614. doi: 10.3389/fpls.2025.1729614 (PMC12816331; doi:10.3389/fpls.2025.1729614)
Supplement: Supplementary file 1 [file Table1.docx]

Supplementary Material

# Supplementary Table 1. Mean monthly air temperature (AirT), relative humidity (RH), and Photosynthetically Active Radiation (PAR) within the polytunnel during the experimental period from April to December 2023. Daytime values cover the 12-hour period from 07:30 to 19:30. The standard error of the mean (SEM) is indicated by the ± values and n is the number of daily observations.

| **Month** | **Day/Night** | **AirT (°C)** | **RH (%)** | **PAR (µmol m^-2^s^-1^)** | **n** |
| --- | --- | --- | --- | --- | --- |
| Apr | Day | 18.45 ±0.87 | 58.18 ±2.99 | 437.80 ± 33.88 | 28 |
| Apr | Night | 8.74 ±0.39 | 83.13 ±1.29 | 3.37 ± 0.42 | 28 |
| May | Day | 21.17 ±0.54 | 53.92 ±2.02 | 625.33 ± 32.33 | 31 |
| May | Night | 11.42 ±0.29 | 85.34 ±0.71 | 19.47 ±1.53 | 31 |
| Jun | Day | 24.40 ±0.61 | 53.64 ±3.00 | 664.87 ±44.83 | 30 |
| Jun | Night | 15.63 ±0.46 | 82.65 ±129 | 27.91 ±1.96 | 30 |
| Jul | Day | 20.22 ±042 | 65.89 ±213 | 490.09 ±31.04 | 31 |
| Jul | Night | 15.38 ±0.31 | 84.70 ±1.32 | 17.18 ±1.58 | 31 |
| Aug | Day | 20.85 ±0.51 | 66.07 ±1.59 | 414.05 ±23.08 | 31 |
| Aug | Night | 15.37 ±0.27 | 86.50 ±0.63 | 6.40 ±0.75 | 31 |
| Sep | Day | 21.55 ±0.75 | 69.08 ±2.22 | 290.85 ±28.35 | 30 |
| Sep | Night | 15.15 ±0.40 | 89.11 ±0.55 | 0.44 ±0.13 | 30 |
| Oct | Day | 16.40 ±0.56 | 77.19 ±1.74 | 150.31 ±12.29 | 31 |
| Oct | Night | 11.50 ±0.52 | 90.47 ±0.61 | 0.00 ±0.00 | 31 |
| Nov | Day | 9.97 ±0.51 | 85.82 ±1.11 | 64.62 ±4.65 | 30 |
| Nov | Night | 7.76 ±0.58 | 91.63 ±0.43 | 0.00 ±0.00 | 30 |
| Dec | Day | 6.51±1.03 | 92.15 ±1.00 | 33.06 ± 3.09 | 14 |
| Dec | Night | 4.75 ±1.02 | 93.33 ±0.60 | 0.00 ±0.00 | 15 |

**
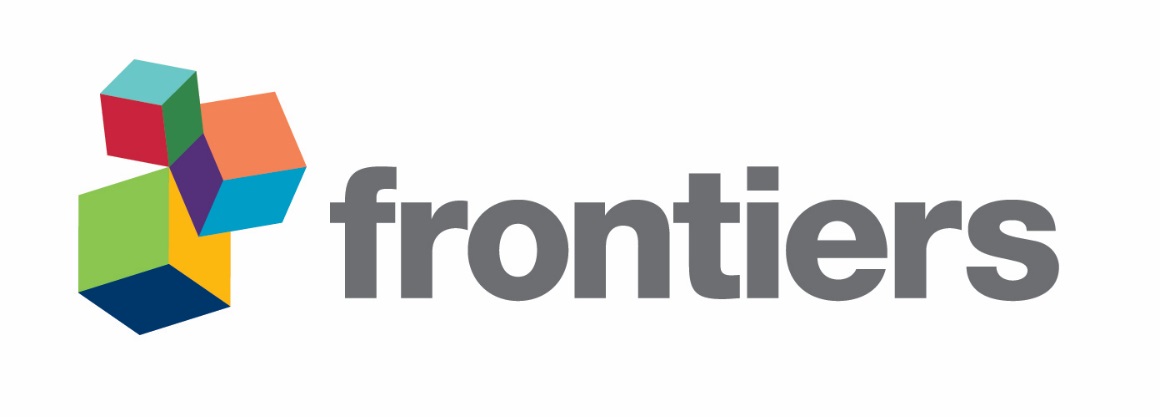
**
